# Beyond the psoas: iliopsoas abscess with thigh extension successfully managed by percutaneous approach

**DOI:** 10.1093/jscr/rjaf792

**Published:** 2025-10-10

**Authors:** Yavor Asenov, Ivan Valentinov Dimitrov, Mariyan Kotsev, Todor Angelov, Branimir Golemanov, Plamen Getsov, Nikolay Penkov

**Affiliations:** Department of Surgery, Medical University – Sofia University General Hospital “Tsaritsa Yoanna – ISUL”, Sofia 1527, Bulgaria; Department of Surgery, Medical University – Sofia University General Hospital “Tsaritsa Yoanna – ISUL”, Sofia 1527, Bulgaria; Department of Gastroenterology, Medical University – Sofia University General Hospital “Tsaritsa Yoanna – ISUL”, Sofia 1527, Bulgaria; Department of Gastroenterology, Medical University – Sofia University General Hospital “Tsaritsa Yoanna – ISUL”, Sofia 1527, Bulgaria; Department of Gastroenterology, Medical University – Sofia University General Hospital “Tsaritsa Yoanna – ISUL”, Sofia 1527, Bulgaria; Department of Diagnostic Imaging, University General Hospital “Tsaritsa Yoanna – ISUL” Medical University – Sofia, Sofia 1527, Bulgaria; Department of Surgery, Medical University – Sofia University General Hospital “Tsaritsa Yoanna – ISUL”, Sofia 1527, Bulgaria

**Keywords:** iliopsoas abscess, thigh extension, percutaneous drainage, image-guided intervention, *Staphylococcus aureus*

## Abstract

Iliopsoas abscess is an uncommon but potentially life-threatening condition. Distal extension into the thigh is extremely rare and usually requires surgery. We report the case of a 65-year-old woman presenting with fever, back pain, and impaired hip mobility. Contrast-enhanced computed tomography revealed a multiloculated iliopsoas abscess extending into the thigh adductor compartment. Under combined ultrasound and fluoroscopic guidance, multi-access percutaneous drainage was performed using one retroperitoneal and two femoral catheters, yielding purulent material positive for *Staphylococcus aureus*. Targeted antibiotic therapy and serial catheter lavages led to rapid recovery. Drains were removed after 14 days, and the patient remained symptom-free at 3-month follow-up. This case demonstrates that even complex iliopsoas abscesses with thigh extension can be successfully treated with a tailored image-guided percutaneous approach, providing a safe and effective alternative to open surgery in selected cases.

## Introduction

Iliopsoas abscess (IPA) is an uncommon but serious condition, with an annual incidence of less than one case per 100 000 population [1]. Clinical presentation is often nonspecific, and the classic triad of fever, flank pain, and hip limitation is present in only one-third of patients [1, 2], frequently causing diagnostic delay. Computed tomography (CT) remains the diagnostic gold standard, while ultrasound provides real-time guidance for drainage when feasible [1–3]. Etiologically, IPAs are classified as primary, most often hematogenous and due to *Staphylococcus aureus* [4], or secondary, usually polymicrobial and arising from gastrointestinal or genitourinary pathology [1, 2]. Most cases are confined to the retroperitoneum; distal extension beyond the psoas sheath, particularly into the thigh, is exceedingly rare and usually requires surgical drainage [5, 6]. We present such a case successfully treated with a tailored percutaneous approach.

## Case report

A 65-year-old female presented with severe general deterioration, high-grade fever, low back pain, and a forced flexion posture of the left hip. Her medical history included gastroesophageal reflux disease, erosive gastritis, hemorrhoidal disease, arterial hypertension, type 2 diabetes, previous mitral and aortic valve replacements, cholecystectomy, and total hysterectomy for cervical carcinoma, followed by radiotherapy. Laboratory tests revealed leukocytosis (16 × 10^9^/l) and elevated C-reactive protein (246 mg/l).

**Figure 1:**
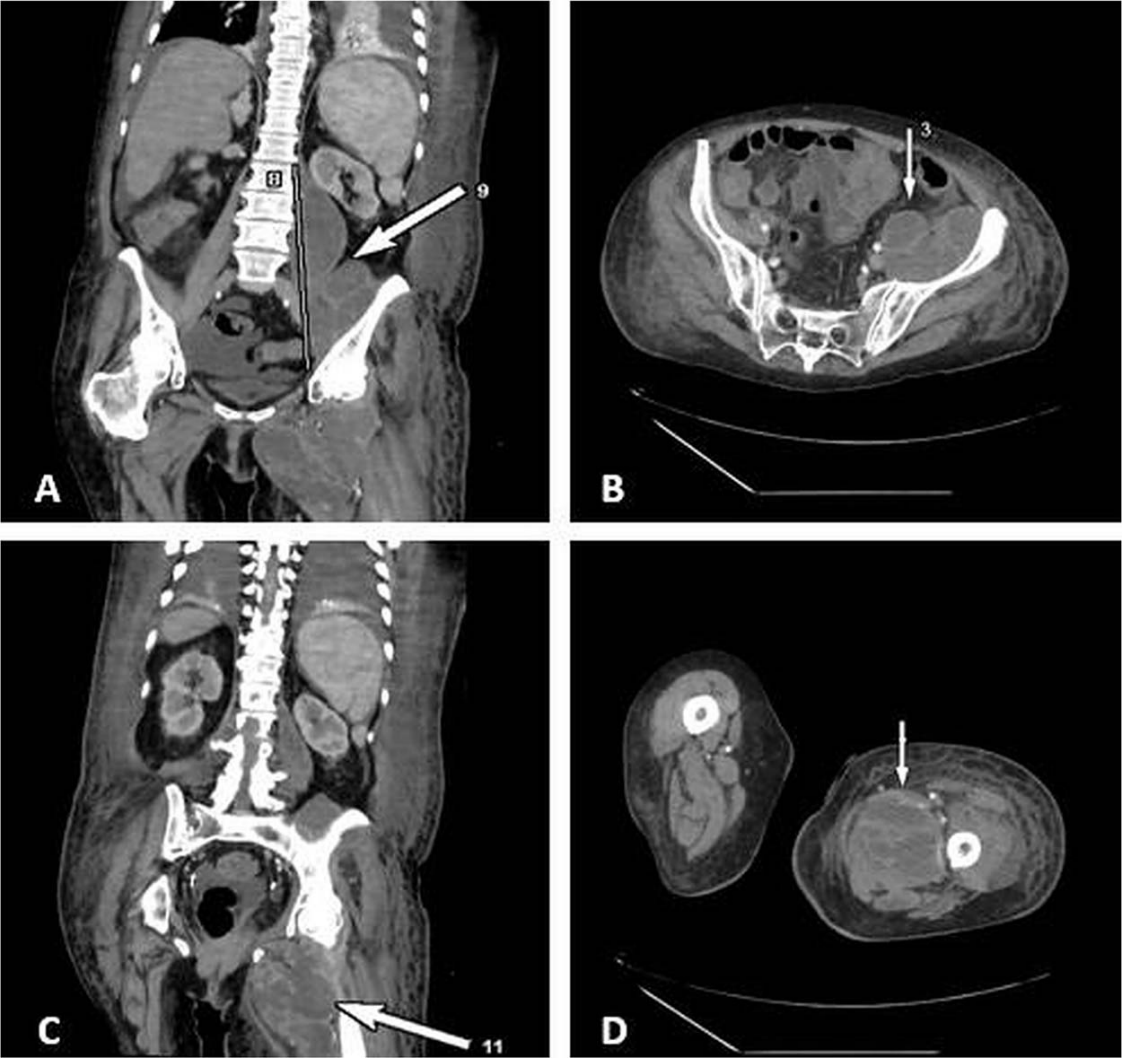
CT demonstrates multiloculated IPA with distal extension into the thigh adductor compartment. (A) Coronal CT showing a multiloculated IPA. (B) Axial CT demonstrating the psoas collection. (C) Coronal CT illustrating distal extension along the psoas sheath into the adductor compartment. (D) Axial CT of the thigh confirming distal involvement.

Contrast-enhanced CT demonstrated a large multiloculated IPA extending distally along the psoas sheath into the upper thigh, involving the adductor compartment (Fig. 1).

**Figure 2:**
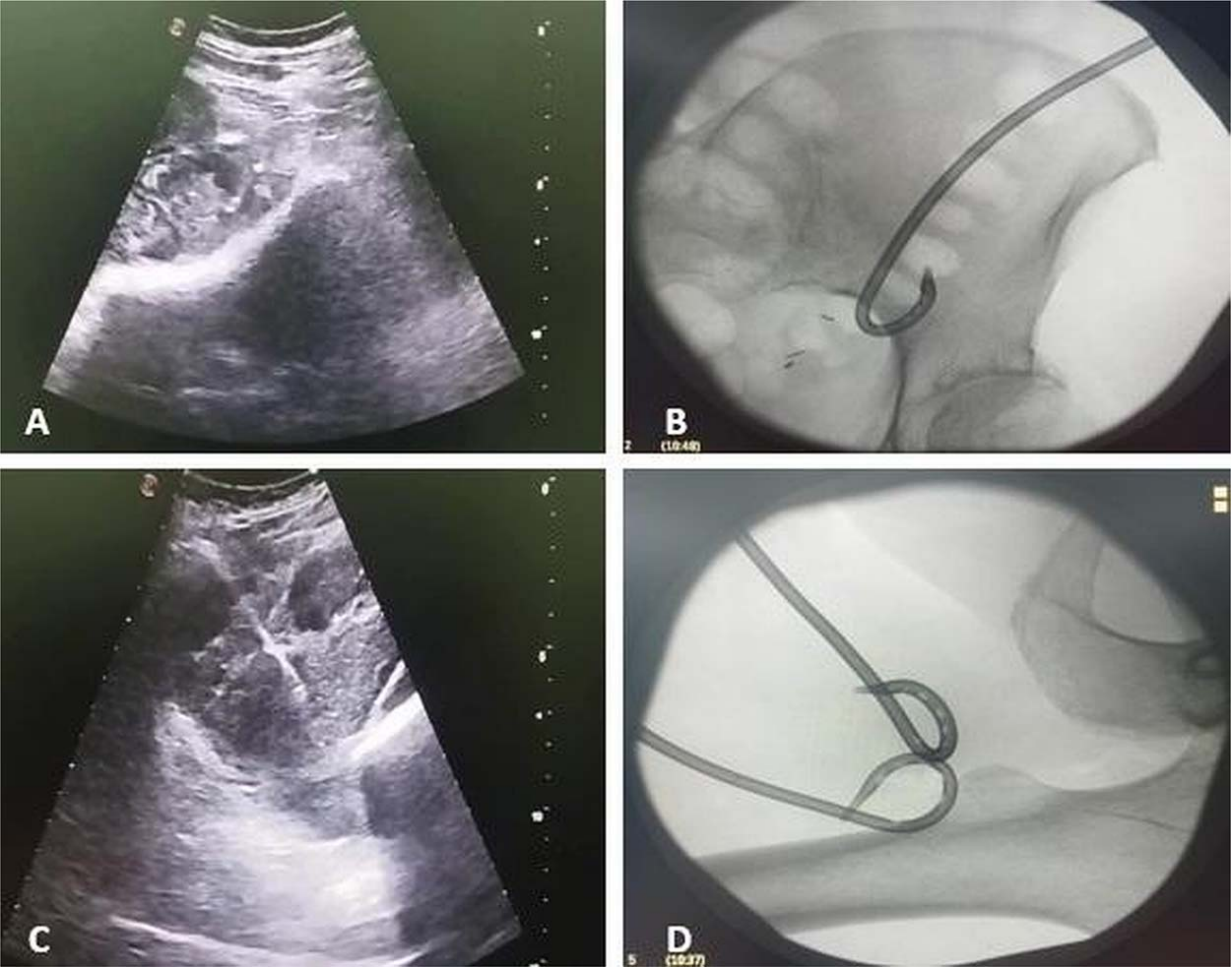
Ultrasound and fluoroscopy show multi-access percutaneous drainage with one retroperitoneal and two femoral catheters. (A) Ultrasound-guided retroperitoneal puncture into the psoas collection. (B) Fluoroscopic image demonstrating catheter placement in the psoas component. (C) Ultrasound-guided puncture of the thigh extension. (D) Fluoroscopic confirmation of one retroperitoneal and two femoral drains in situ.

**Figure 3:**
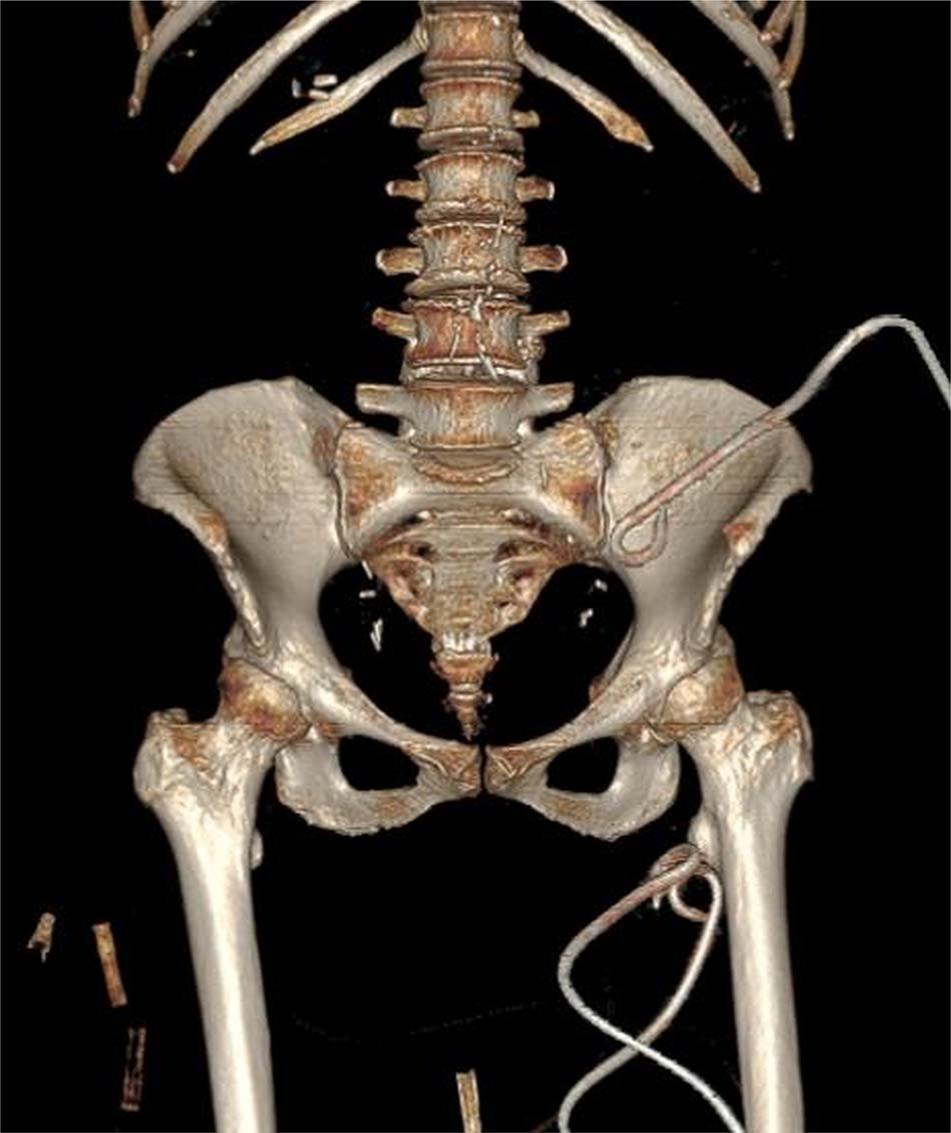
3D CT reconstruction depicting the final positions of the drainage catheters.

Due to the complex anatomy, a single access was deemed insufficient. Under real-time ultrasound and fluoroscopic guidance, a retroperitoneal approach was first used: an 18-G needle and guidewire were advanced in the left midaxillary line parallel to the iliac crest, followed by tract dilation and placement of a 12-G pigtail catheter into the psoas component. Subsequently, two additional 12G catheters were placed percutaneously via a femoral approach into the thigh extension (Fig. 2). In total, ~200 ml of purulent material was drained. The localization of the drains was confirmed by subsequent CT (Fig. 3).

The patient’s clinical condition improved rapidly: fever resolved within 72 hours, inflammatory markers normalized, and she regained the ability to extend her leg almost immediately after drainage. Serial lavages with sterile saline were performed through all catheters to enhance clearance of debris and reduce pus viscosity.

Microbiological analysis identified *S. aureus* in both abscess fluid and blood cultures. Empirical treatment with metronidazole and cefoperazone/sulbactam was initiated and subsequently tailored to culture results with the addition of vancomycin.

By day 14, the catheters were gradually removed after cessation of purulent drainage and reduction of output to <10 ml over 24 hours. The patient was discharged in good condition and remained symptom-free at the 3-month follow-up.

## Discussion

Management of IPA has shifted from open surgery to image-guided percutaneous drainage, which achieves clinical success in 70%–90% of cases, with mortality generally <5% [7, 8]. Antibiotics alone have high failure rates in larger or multiloculated collections [1, 2]. Predictors of percutaneous failure include multiloculation, viscous pus, gas-forming organisms, and anatomically inaccessible collections [9].

Mortality still ranges from 5% to 15%, rising above 40% with delayed diagnosis or septic shock [2, 10]. Septic shock occurs in up to 20% of cases, and overall in-hospital mortality is 10%–12% in recent series [2, 10]. Our case was consistent with a primary abscess caused by *S. aureus*, the most frequent organism in such presentations [4].

Complications of percutaneous drainage are relatively infrequent, including bleeding (≤5%), fistula formation (3%–5%), and injury to adjacent structures (≤2%); careful planning and structured catheter management reduce these risks [3]. Imaging guidance is crucial: CT delineates the full extent of disease and allows trajectory planning, ultrasound enables safe puncture and catheter placement, and fluoroscopy assists in abscessography and catheter navigation. Combined use of these modalities, although not routine, can enhance precision in challenging cases [3, 7, 8].

Distal extension of IPA into the thigh is exceedingly rare, with most reported cases managed surgically [5, 6]. Our case suggests that even such complex presentations may be managed by a carefully tailored percutaneous strategy. Multi-access drainage with adjunctive lavages allowed effective clearance and rapid recovery, avoiding the need for open surgery in selected patients.
